# Residual Neuromuscular Blockade and Postoperative Pulmonary Complications in the Post-anesthesia Care Unit: A Prospective Observational Study

**DOI:** 10.7759/cureus.51013

**Published:** 2023-12-23

**Authors:** Buthaina A. Bucheery, Hasan M Isa, Owais Rafiq, Nada Abdulrahman Almansoori, Zaina Abdulsatar Abdul Razaq, Zeana A. Gawe, Jihad Ali Almoosawi

**Affiliations:** 1 Department of Anesthesia, Salmaniya Medical Complex, Manama, BHR; 2 Department of Pediatrics, Arabian Gulf University, Manama, BHR; 3 Department of Pediatrics, Salmaniya Medical Complex, Manama, BHR

**Keywords:** post-anesthesia care unit, neuromuscular monitoring, airway support, train-of-four, postoperative complications, residual block

## Abstract

Background

Neuromuscular blocking agents (NMBAs) are employed during general anesthesia induction for endotracheal intubation and to facilitate specific surgeries requiring muscle relaxation. However, residual neuromuscular blockade (RNMB) can lead to respiratory complications in post-anesthesia care units (PACUs). This study investigates RNMB incidence in PACUs and its association with postoperative airway and respiratory issues.

Methods

A prospective observational study on patients undergoing general anesthesia with NMBAs was conducted at the Department of Anesthesia, Salmaniya Medical Complex, Bahrain, over six months (April to September 2023). Train-of-four (TOF) ratios were calculated using an acceleromyograph upon PACU arrival. Data on demographics, perioperative variables, and postoperative complications were recorded.

Results

Among 82 patients, 30 (36.6%) had RNMB upon PACU arrival. RNMB incidence declined: 17.1% at 10 minutes, 6.1% at 20 minutes, and 2.4% at 30 minutes, resolving by 40 minutes. Demographics and procedure duration showed no correlation with RNMB. Postoperative respiratory complications affected 23.2% of patients, notably higher in those with RNMB (p = 0.001). Among patients with TOF <90% at PACU arrival, 46.7% experienced complications compared to 9.6% with TOF ≥90% (p<0.001). Participants without RNMB had a significantly higher weight (p = 0.046). Airway support was required for 30% of patients, all with TOF <90% (p<0.001).

Conclusion

This study emphasizes the importance of assessing and monitoring neuromuscular function to detect and prevent RNMB in PACUs. RNMB presence correlated with an increased susceptibility to postoperative respiratory complications. Regular quantitative neuromuscular monitoring is advisable in clinical practice to proactively mitigate RNMB incidence and its complications.

## Introduction

Neuromuscular blocking agents (NMBA) serve to aid endotracheal intubation in general anesthesia induction and enhance muscle relaxation in specific surgical procedures [1-3]. These agents competitively antagonize acetylcholine nicotinic receptors at the neuromuscular junction, disrupting neuromuscular transmission [1].

Postoperative residual neuromuscular blockade (RNMB), characterized by a train-of-four (TOF) ratio below 0.7, is linked to postoperative respiratory complications and various postoperative issues [4, 5]. Such complications encompass upper airway muscle weakness, airway obstruction, impaired pharyngeal function, increased aspiration risk, inadequate respiratory function recovery, and compromised hypoxic ventilatory response [4]. Recent investigations have connected postoperative RNMB with a TOF ratio under 0.9 to postoperative respiratory complications [6]. The presence of postoperative respiratory complications significantly impacts the overall postoperative outcome; potentially resulting in prolonged recovery room stays [4].

Quantitative neuromuscular blockade assessment entails observing twitch fade via a TOF stimulation involving four sequential electrical nerve stimuli and evaluating their effects on the corresponding muscle [7].

In this prospective observational study, we aim to assess the incidence of postoperative RNMB in the post-anesthesia care unit (PACU) and its correlation with postoperative airway and respiratory complications. These include the requirement for airway support, incidence of hypoxia, and time to meet discharge eligibility from the PACU. Furthermore, the study endeavors to delve into the relationship between RNMB and the aforementioned complications.

## Materials and methods

Study design and study participants

An observer-blinded prospective observational study was done within a tertiary care center's PACU, at the Department of Anesthesia, Salmaniya Medical Complex for a period of six months from April to September 2023. A total of 82 patients were enrolled in the investigation, with recruitment contingent upon the investigator's presence in the PACU during the study period. This study was approved by the Institutional Ethics Committee, Research Committee for Government Hospitals, at the Department of Anesthesia, Salmaniya Medical Complex, Bahrain (IRB approval number is 21070323, approval date March 7, 2023).

Inclusion criteria encompassed patients falling within the American Society of Anesthesiologists (ASA) classification classes 1, 2, and 3, aged between 18 and 80 years, who had undergone general anesthesia employing an NMBA. The anesthesiologists responsible for administering anesthesia remained unaware of the patients' involvement in the study. Anesthesia conduct, inclusive of muscle relaxant selection and neuromuscular monitoring utilization, was determined at the discretion of the anesthesiologist. Most operating rooms were equipped with peripheral nerve stimulators suitable for monitoring the TOF. To maintain the blinding of anesthetists to their participation, we refrained from observing the use or interpretation of train of four ratios (TOFRs) during surgery. All patients underwent extubation in the operating room before transfer to the PACU.

The primary outcome variable of interest was postoperative RNMB, defined as TOFRs below 0.9 during the patient's PACU stay. Upon PACU admission, two cutaneous electrodes were affixed over the ulnar nerve, followed by the application of a 30mA submaximal stimulus in a TOF configuration. The motor response at the adductor pollicis muscle was quantified using a TOF guard monitor to assess the TOFR. The utilization of submaximal stimuli, validated for patient comfort, was deemed precise in measuring TOFRs within this context. Measurements were repeated every 10 minutes until achieving a TOF exceeding 0.9.

Data collection

To support our exploration of RNMB and postoperative pulmonary complications in the PACU, we employed a data collection sheet to gather patient-specific information. This sheet encompassed a range of variables related to patients, the anesthesia procedure, and their postoperative progress. The variables included patient age, sex, and the American Society of Anesthesiologists classification for physical status (ASA-PS), weight, NMBA dosage, increments, and infusions, as well as the procedure's duration. Vital signs, such as blood pressure, heart rate, and oxygen saturation, were meticulously recorded. Additionally, the data collection sheet documented TOF measurements at time points: 0, 10, 20, 30, and 40 minutes. Any respiratory complications encountered by the patients were also duly noted.

Data analysis

Each patient had an associated data collection sheet encompassing all relevant variables. Data entry was performed using Microsoft Excel (Microsoft® Corp., Redmond, WA USA), with subsequent analysis conducted through the utilization of Statistical Package for Social Sciences (SPSS) version 24 (IBM Corp., Armonk, NY, USA). Descriptive statistics, including mean, median, standard deviation, and interquartile range (IQR), were computed for quantitative variables. For qualitative variables, numbers and percentages were tabulated. Bivariate analysis involved the utilization of the Chi-square test and Fisher's exact test for qualitative variables and the Student's unpaired t-test and Mann-Whitney U test for quantitative variables. A significance level of p < 0.05 was employed across all statistical tests.

## Results

Over a two-month span, we evaluated 82 patients who met our inclusion criteria upon admission to the PACU. The demographic characteristics of the screened cohort are summarized in Table 1. The mean age of these individuals stood at 40.9 ± 14.9 years. Females constituted 56.1% (n=46) of the total, whereas males accounted for 43.9% (n=36). Predominantly, patients fell into ASA-PS classes I (51.9%) and II (45.7%), with a minor portion (2%) categorized as ASA-PS class III. The mean weight of the patients was 81.5 ± 19.9 kg. Intraoperatively, all patients received cisatracurium as their NMBA at a median dose of 10 mg (IQR: 8-12). A notable 57.3% (n=47) of patients received one or more increments of NMBA during the intraoperative phase, while only two (2.4%) individuals received NMBA infusions. To reverse neuromuscular blockade, 81 (98.8%) patients received an anticholinesterase agent (neostigmine). The median duration of the procedures was 90 (IQR: 84-95) minutes. It's noteworthy that neuromuscular monitoring was not employed before extubation for any of the patients.

**Table 1:** Patients' demographics in relation to intraoperative course and residual neuromuscular blockade The data were represented as mean ± SD, n (%) and median, interquartile range (IQR). The comparison was done between RNMB and no RNMB. ^a ^Student t-test; ^b ^Fishers’ exact test; ^c ^Pearson chi-square; ^d ^Mann-Whitney U test ^*^denotes significant p<0.05, ^†^denotes non-significant p>0.05. RNMB: residual neuromuscular block; TOF: train-of-four; SD: standard deviation; IQR: interquartile range; ASA-PS class: American Society of Anesthesiologists Physical Status class; NMBD: neuromuscular blocking drug.

Variables	Total n=82 (100)	RNMB (TOF ratio < 90), n = 30 (36.6)	No RNMB (TOF ratio > 90), n = 52 (63.4)	P value
Age in years, mean ± SD	40.9 ± 14.9	39.5 ± 13.3	41.6 ± 15.9	0.537^a^^†^
Sex, n (%)				0.818^b^^†^
Male	36 (43.9)	14 (46.7)	22 (42.3)	
Female	46 (56.1)	16 (53.3)	30 (57.7)	
ASA-PS class (n=81)				0.428^c^^†^
I	42 (51.9)	17 (58.6)	25 (48.1)	
II	37 (45.7)	12 (41.4)	25 (48.1)	
III	2 (2.5)	0 (0.0)	2 (3.8)	
Weight (kg), mean ± SD (n=79)	81.5 ± 19.9	75.3 ± 20.1	84.7 ± 19.2	0.046^a^^*^
NMBD induction dose (mg), median (IQR)	10 (8-10)	10 (8-10)	10 (8-10)	0.576^d^^†^
Increments of NMBD	47 (57.3)	20 (66.7)	27 (51.9)	0.249^b^^†^
NMBD infusion intraoperatively	2 (2.4)	1 (3.3)	1 (1.9)	1.000^b^^†^
Reversal used (n=81)				1.000^b^^†^
1	80 (98.8)	29 (100.00)	51 (98.1)	
1.5	1 (1.2)	0 (0.0)	1 (1.9)	
Duration of procedure in minutes, median (IQR)	90 (60-120)	90 (60-120)	112.5 (60-120)	0.549^†^

On arrival at the PACU, 30 (36.6%) patients were found to have TOF less than 90% which reflects RNMB, while 52 patients (63.4%) had TOF more than or equal to 90% (Table 1). Patients with RNMB had significantly lower mean weight than those without RNMB (p=0.046). It is shown that there is no significance of the age, the sex, or the ASA-PS class of the patients in relation to postoperative RNMB. There was no statistical significance with regard to age, sex, weight or the duration of the procedure on RNMB. The utilization of neuromuscular agent doses, increments, or infusions did not exhibit any discernible correlation with the occurrence of RNMB. Similarly, the procedural duration did not display a significant relationship with the presence of RNMB.

**Figure 1:**
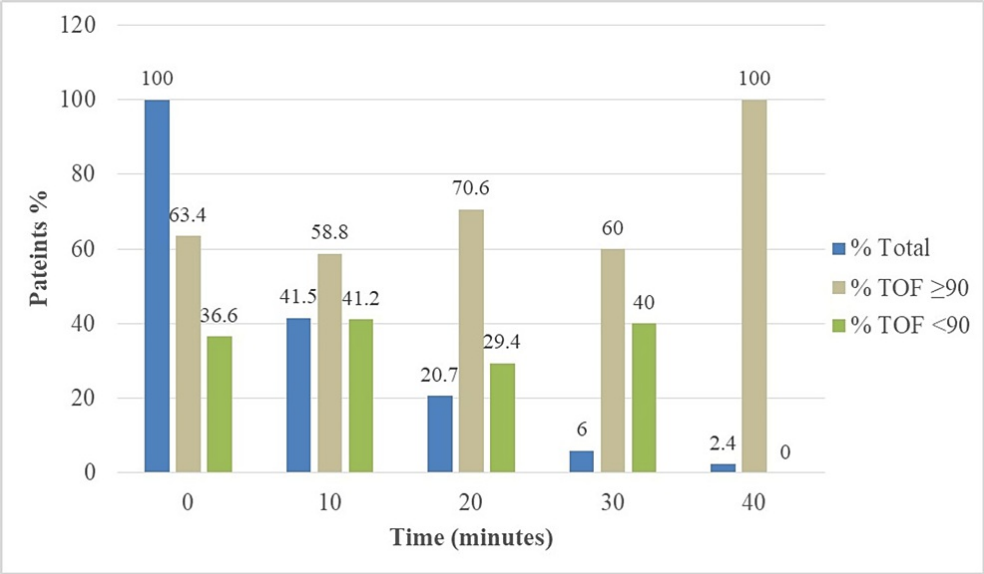
Patients with residual neuromuscular blockade at different time intervals TOF: Train-of-four

As illustrated in Figure 1, approximately one-third (36.6%) of patients demonstrated RNMB upon their arrival in the PACU. Subsequently, 17.1% (n=14) of patients exhibited RNMB at 10 minutes into their PACU stay, followed by 6.1% at the 20-minute mark, and 2.4% at the 30-minute point. Notably, all patients had fully resolved their neuromuscular blockade (TOF ≥90%) by the 40-minute mark of their PACU stay.

Postoperative respiratory complications afflicted 19 (23.2%) patients, with 14 (17.1%) of them displaying a TOF value below 90% upon PACU admission, in contrast to only 5 (6.1%) patients with a TOF value of ≥90% (p < 0.001). Among the 9 (10.9%) patients requiring airway support, all presented with a TOF value below 90% on PACU arrival (p < 0.001). Furthermore, 8 (9.8%) patients manifested signs of respiratory distress, characterized by a respiratory rate exceeding 22 breaths per minute, while 2 (2.4%) patients experienced hypoxemia. An additional 3 (3.7%) patients encountered upper airway weakness, leading to difficulties in swallowing or speaking. Detailed data are available in Table 2.

**Table 2:** Comparison between postoperative respiratory complications and Train-Of-Four value at Zero minute The data were shown as n (%). The comparison between RNMB and no RNMB was done using Fisher’s exact test. ^*^ denotes significant p<0.05, ^†^ denotes non-significant p>0.05. TOF: train of four; RNMB: residual neuromuscular block; RR: respiratory rate; SpO2: blood oxygen saturation.

Variables	Total n = 82 (100)	RNMB (TOF ratio < 90), n = 30 (36.6)	No RNMB (TOF ratio > 90), n = 52 (63.4)	P value
Hypoxemia (SpO_2_ <93%)	2 (2.4)	2 (6.7)	0 (0.0)	0.131^†^
Signs of respiratory distress (RR >20)	8 (9.8)	3 (10.0)	5 (9.6)	1.000 ^†^
Upper airway obstruction	9 (11)	9 (30.0)	0 (0.0)	<0.001^*^
Upper airway muscle weakness (Complain by patient)	3 (3.7)	3 (10.0)	0 (0.0)	0.046^†^
Total number of patients who developed one or more respiratory complication	19 (23.2)	14 (46.7)	5 (9.6)	<0.001^*^

## Discussion

This study finding illuminates a critical association, revealing that patients exhibiting RNMB upon PACU arrival are disproportionately prone to experiencing adverse respiratory events. The rapid resolution of RNMB within 40 minutes accentuates the importance of vigilant monitoring during this temporal window. Furthermore, demographic factors and the dosage of neuromuscular agents did not significantly correlate with RNMB, suggesting a complex interplay of variables.

Incidence of postoperative RNMB

The investigation into RNMB within the PACU revealed intriguing insights into its incidence and temporal resolution. The study revealed that 36.6% of patients exhibited RNMB in the PACU, underscoring the prevalence of this condition and its clinical relevance. Moreover, a comprehensive examination of the time-based progression of RNMB at 10, 20, and 30 minutes unveiled a striking trend. A substantial proportion of patients displayed RNMB immediately upon PACU admission, but as time elapsed, the resolution of RNMB was remarkably rapid. Most patients achieved TOF ratio exceeding 0.9 within 40 minutes. This temporal aspect of RNMB resolution highlights the dynamic nature of neuromuscular recovery postoperatively.

The clinical significance of our findings cannot be overstated. RNMB, as indicated by the persistence of low TOF ratios, has been associated with various adverse events in the literature, including postoperative respiratory complications. Similar studies [8-14] in the past emphasize the clinical importance of RNMB detection, as its presence has been linked to factors such as age, type of neuromuscular agents used, and the choice of reversal agents. Esteves et al.'s study [8] highlights the importance of RNMB detection with a 5.5% incidence rate upon PACU arrival, suggesting that even a modest incidence warrants clinical attention. The temporal resolution observed in our study further accentuates the urgency of vigilant monitoring during this critical window of time. Our study's findings underscore the prevalence of RNMB in the PACU and its temporal dynamics, emphasizing the need for meticulous monitoring and timely interventions to mitigate its clinical impact.

Patient demographics

The patients’ mean ± SD age, as indicated in this study, was 40.9 ± 14.9 years and aligns with the findings of Carrillo-Torres et al. [11] where they found that older adults had a higher odds ratio for RNMB. In our study, female predominance was noted accounting for 56.1% and 43.9% being males. This sex-related distribution is in line with the observation made by Alenezi et al. [12], where they found that RNMB was higher in females.

Regarding the ASA-PS class, our study revealed that most patients fell into ASA-PS class I (51.9%) and class II (45.7%), with a smaller percentage classified with ASA-PS class III (2%). Interestingly, this distribution parallels the findings in Belcher et al.'s study [10], where they reported that intensive care unit admission rates were notably higher in those patients with any major complications compared to those without, underscoring the relevance of ASA-PS class in the context of RNMB and its clinical implications.

The study also recorded the weight of patients, and it's noteworthy that the weight distribution was relatively balanced among different categories. Our results indicated that no demographic factors, including age, sex, or ASA-PS class, were significantly associated with the incidence of RNMB (except for the weight) consistent with Lin et al.'s findings [9] that highlighted the multifactorial nature of RNMB reinforcing the notion that clinical outcomes are influenced by a complex interplay of factors.

Analysis of patient demographics in our study elucidates the characteristics of the sample while aligning with and complementing findings from prior research. The absence of significant associations between demographic factors and the incidence of RNMB underscores the multifaceted nature of this phenomenon, substantiating the importance of monitoring and intervention strategies in mitigating its clinical impact.

Neuromuscular blocking agent usage

Our exploration into the usage of NMBA within the study sample elucidated several noteworthy observations. The median dose of cisatracurium, which stood at 10 mg, indicates a standard dosage employed during the intraoperative period. This finding aligns with the observations in Alenezi et al.'s study [12], where 76.7% of patients received neostigmine as a reversal drug, emphasizing the commonality of these practices. Furthermore, a substantial proportion of patients (57.3%) in our study received additional increments of NMBA during the intraoperative period. Interestingly, this practice did not significantly correlate with the incidence of RNMB, as established in our results. This observation resonates with Batistaki et al.'s study [15], which detected no significant difference in RNMB between patients who received neostigmine and those who were administered sugammadex. These findings collectively underscore the complex relationship between NMBA usage and RNMB. Moreover, our study highlighted the minimal use of NMBD infusion during the procedure, with only two (2.4%) patients receiving such treatment. This practice concurs with the study by Esteves et al. [8], where 96.2% got reversal agent, with 96.6% of those using sugammadex. The parallel observations indicate a preference for timely reversal of neuromuscular blockade in clinical settings [15-19].

The uniform application of anticholinesterase (neostigmine) as a reversal agent in our study further accentuates the consistency of this practice, aligning with the results from Esteves et al. and Raval et al. [8, 20]. However, the dose and usage of NMBA did not exhibit significant correlations with the incidence of RNMB in our study, as noted earlier. This finding emphasizes the necessity of vigilant monitoring and individualized interventions to manage RNMB effectively.

RNMB and postoperative respiratory complications

The intricate relationship between RNMB and postoperative respiratory complications emerges as a focal point of discussion. The study illustrated that 23.2% of patients experienced postoperative respiratory complications, underscoring the clinical importance of monitoring, and managing RNMB in the PACU.

Our findings unveiled a compelling association between low TOF values upon arrival in the PACU and these postoperative complications. Specifically, patients with TOF values indicative of RNMB were at a heightened risk of experiencing hypoxemia (SpO2 <93%), signs of respiratory distress (RR >20 cycles per minute), upper airway obstruction, and upper airway muscle weakness, which was notably complained of by the patients themselves. These results align with the findings of similar studies [21-25], that measured TOF ratios and assessed RNMB with TOF ratio of less than 0.9 during tracheal extubation, emphasizing the predictive role of TOF values.

The significance of TOF values in predicting postoperative complications is further underscored by the outcomes presented in our study. Those with RNMB are distinctly at higher risk of developing one or more respiratory complications, emphasizing the critical nature of monitoring and intervening in cases of RNMB [26-29]. These findings resonate with Murphy et al.'s study [30], where they reported a substantially higher incidence of PRNB in elderly patients, who concurrently exhibited increased rates of hypoxemic events, airway obstruction, and other respiratory complications, corroborating the link between neuromuscular blockade and adverse outcomes.

Our study elucidates a compelling connection between RNMB and postoperative respiratory complications, reinforcing the clinical relevance of vigilant neuromuscular monitoring. The interplay between low TOF values and the development of these complications accentuates the significance of prompt detection and intervention in mitigating the impact of RNMB on patient outcomes.

Implications of the study

This study's implications are profound in the realm of perioperative care. The clear association between RNMB and respiratory complications in the postoperative period emphasizes the imperative need for vigilant neuromuscular monitoring in the PACU. This study underscores the significance of maintaining optimal TOF values to prevent hypoxemia, respiratory distress, airway obstruction, and muscle weakness. This aligns with existing literature, emphasizing the clinical relevance of preventing RNMB, particularly in the elderly. Timely reversal of neuromuscular blockade, individualized dosing, and tailored monitoring strategies are imperative to enhance patient safety and optimize postoperative outcomes.

Limitations of the study

Several limitations merit consideration in interpreting our study's findings. Firstly, our study's sample size was relatively modest, which may affect the generalizability of results to broader populations. Additionally, the absence of quantitative neuromuscular monitoring before extubation in all patients may introduce variability in detecting RNMB. Furthermore, the observational nature of the study implies that causality cannot be definitively established. Lastly, our study focused on the PACU, but the impact of RNMB may extend into the postoperative period.

## Conclusions

The study illustrates the association between RNMB and respiratory complications in PACU. Our findings emphasize the clinical significance of vigilant neuromuscular monitoring and prompt intervention to maintain optimal TOF values. Weights of the participants were significantly more in those without RNMB. The rapid resolution of RNMB within 40 minutes underscores the potential for timely corrective measures.
